# Development of immune-related adverse events is associated with improved survival outcomes in cancer patients receiving immune checkpoint inhibitors

**DOI:** 10.3389/fimmu.2026.1910109

**Published:** 2026-08-20

**Authors:** Faizah M. Alotaibi, Seham A. Khashwayn, Lamia K. Alshamlani, Rayan A. Almutairi, Anas K. Alghamdi, Nawaf M. Alamer, Mohannad A. Alghamdi, Badi A. Alotaibi, Kanan Alshammari

**Affiliations:** 1 College of Science and Health Professions, King Saud bin Abdulaziz for Health Science, Riyadh, Saudi Arabia; 2 King Abdullah International Medical Research Center, Al-Ahsa, Saudi Arabia; 3 Ministry of National Guard Health Affairs, Riyadh, Saudi Arabia; 4 College of Medicine, King Saud Bin Abdulaziz for Health Science, Riyadh, Saudi Arabia; 5 Emergency Medicine, King Fahad Medical City, Riyadh, Saudi Arabia; 6 Internal Medicine, Department of Medicine, King Faisal Specialized Hospital and Research Center, Riyadh, Saudi Arabia; 7 Department of Clinical Laboratory Sciences, College of Applied Medical Sciences, King Saud Bin Abdulaziz University for Health Sciences, Riyadh, Saudi Arabia; 8 Department of Oncology, Ministry of National Guard Health Affairs, Riyadh, Saudi Arabia

**Keywords:** cancer, ICI, immune-checkpoint blockade, immune-related adverse events, irAE

## Abstract

Immune checkpoint inhibitors (ICIs) have become the standard-of-care in various cancer types. However, treatment with ICIs occasionally cause immune-related adverse events (irAEs). This study assessed the incidence and clinical features of irAEs and examined their association with treatment outcomes in patients with solid tumors receiving ICIs. We conducted a retrospective cohort study including 385 patients with histologically confirmed solid tumors treated with ICIs. Patients were categorized according to the occurrence of irAEs. Baseline clinicopathological characteristics, irAE patterns, management strategies, and survival outcomes were analysed. To minimize immortal time bias, landmark analyses at three months were performed. Multivariable Cox regression models were used to evaluate the association between irAEs occurring within the first three months of treatment and subsequent survival outcomes after adjustment for clinically relevant confounders. Among 385 patients, 181 (47%) developed at least one irAE during ICI treatment. The most frequently observed irAEs included transaminitis, thyroiditis, pneumonitis, rash, and colitis. Patients who developed irAEs had significantly improved survival outcomes compared with those without irAEs. In landmark Kaplan–Meier analyses, patients who developed irAEs during follow-up had significantly improved OS and PFS compared with those who did not. In landmark-adjusted multivariable analysis, irAEs was associated with improved OS (HR 0.673, 95% CI 0.453–1.00; P = 0.0498), whereas the association with PFS was not statistically significant (HR 0.851, 95% CI 0.630–1.149; P = 0.2916). A baseline neutrophil-to-lymphocyte ratio (NLR) <3 was associated with favourable survival outcomes. Most irAEs were managed with corticosteroids and temporary treatment interruption. Development of irAEs was associated with improved survival outcomes in patients with solid tumors treated with ICIs. These findings support the hypothesis that irAEs was associated with enhanced anti-tumor immune activity. Real-world characterization of irAEs and their management may help optimize patient monitoring and treatment strategies during immunotherapy.

## 1 Introduction

Immune checkpoint inhibitors (ICIs) target immune-checkpoint suppressive signalling pathway, which can lead to the initiation of antitumor immune responses, and thus have generated durable clinical benefit across a broad range of malignancies (1). Currently, FDA approved ICIs that target programmed cell death protein 1 (PD-1), cytotoxic T-lymphocyte-associated antigen 4 (CTLA-4), which are expressed on immune cells or programmed death-ligand 1 (PD-L1), which is expressed on tumour cells (2). However, despite these therapeutic advances, the clinical use of ICIs is associated with a unique spectrum of toxicities known as immune-related adverse events (irAEs) (3, 4). Unlike conventional chemotherapy-associated toxicities, irAEs arise from excessive activation of immune system against host organs (5–7). The most commonly involved organs include the skin, gastrointestinal tract, endocrine glands, liver, and lungs, although less frequent but potentially severe cardiovascular, renal, and hematologic toxicities have also been reported (5–8). While many irAEs are manageable, severe toxicities may lead to hospitalization, treatment interruption, discontinuation of ICI, or initiation of immunosuppressive therapy (3, 9). However, beyond their clinical significance as treatment-related toxicities, irAEs have increasingly attracted attention as potential biomarkers of ICI efficacy. Several studies have suggested that patients who develop irAEs experience improved overall survival (OS) and progression-free survival (PFS), potentially reflecting a more robust and sustained systemic immune activation capable of facilitating both anti-tumor effects and autoimmune toxicities (10, 11). This observation has led to the hypothesis that the occurrence of irAEs may represent a clinically observable biomarker of effective anti-tumor immunity. However, currently available evidence remains inconsistent, with substantial heterogeneity across studies regarding the magnitude and direction of these associations (12–15).

Considering the diverse mechanisms of action of ICIs, the substantial heterogeneity of irAEs severity, timing of onset, and incidence is not surprising. Interpatient and organ-specific involvement further contribute to this heterogeneity, with genetic and environmental differences among patients potentially predisposing to subclinical immune responses in specific organs that are subsequently triggered by ICI therapy (16–18). Furthermore, ICIs may be combined with different ICIs and/or with other anticancer treatments which can contribute to conflicting results across the literature. For instance, treatment with anti-PD-1/PD-L1 agents is generally associated with a lower incidence of irAEs compared with anti-CTLA-4 therapy, whereas combined anti-PD-1 and anti-CTLA-4 treatment is associated with the highest risk of irAE occurrence (19).

Management of irAEs represents another important clinical challenge. Corticosteroids remain the major treatment for moderate-to-severe irAEs, while additional immunosuppressive therapies may be required in refractory cases (20). Although these interventions are essential for toxicity control, concerns remain regarding their potential impact on the anti-tumor efficacy of ICIs. Previous studies have reported conflicting findings, with some suggesting that systemic immunosuppression may attenuate ICI benefit (21), whereas others have demonstrated minimal or no effect on survival outcomes (22). Investigating these relationships is increasingly important as the use of ICIs continues to expand in routine oncology practice. Furthermore, Interpretation of prior findings is complicated by several methodological limitations. Many retrospective studies evaluating the prognostic impact of irAEs are susceptible to immortal-time bias (23), as patients must survive long enough during treatment to develop toxicities. Inadequate adjustment for this bias may overestimate the survival advantage associated with irAEs. Most available evidence regarding irAEs originates from clinical trials conducted in highly selected patient populations or from retrospective cohorts derived predominantly from Western and East Asian populations (24). Data from Middle Eastern populations remain limited despite potential differences in patient demographics, comorbidities, tumor characteristics, and treatment patterns that may influence both toxicity profiles and clinical outcomes (25). In addition, few studies have comprehensively evaluated the incidence, clinical characteristics, management strategies, and survival impact of irAEs while accounting for time-dependent bias (26). Therefore, the present study aimed to evaluate the incidence, types, management, and prognostic impact of irAEs in patients with solid tumors treated with ICIs. We further investigated the association between irAE development and survival outcomes using landmark and multivariable analyses to minimize potential time-dependent bias and identify independent predictors of OS and PFS.

## 2 Materials and methods

### 2.1 Study design and patient population

**Figure 1:**
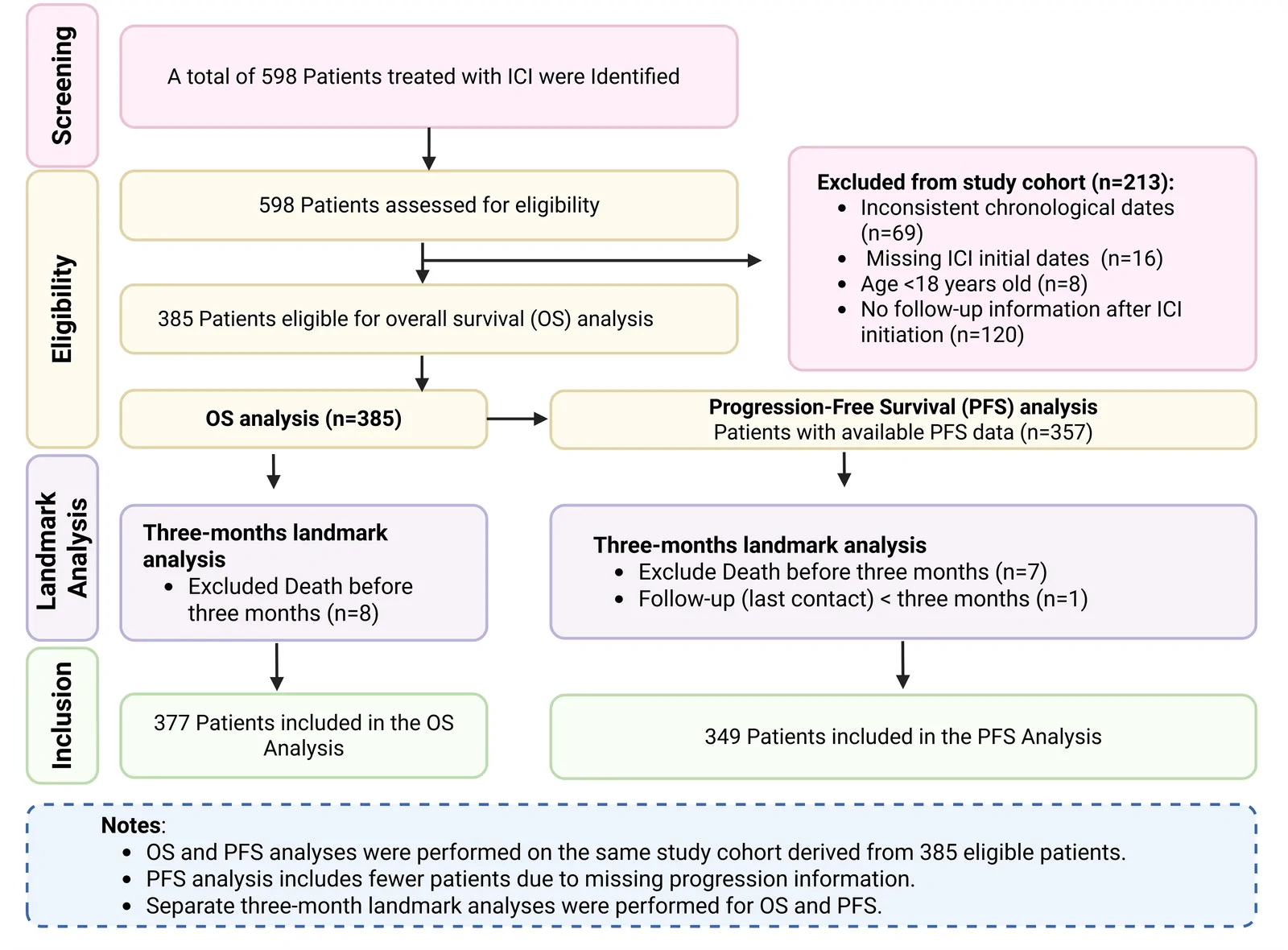
Study flow diagram illustrating patient selection and landmark analysis for OS and PFS. A total of 598 patients treated with ICIs were screened, of whom 385 met the eligibility criteria of the study cohort. Separate analyses were performed for OS and PFS. The PFS cohort included only patients with available progression data (n = 357), whereas the OS analysis included all eligible patients (n = 385). Independent three-month landmark analyses were subsequently conducted for each endpoint. Patients who experienced the corresponding event before the three-month landmark or had insufficient follow-up were excluded, resulting in final landmark cohorts of 377 patients for the OS analysis and 349 patients for the PFS analysis.

This retrospective cohort study included adult patients with histologically confirmed solid tumors who received ICIs at King Abdulaziz Medical City, National Guard Health Affairs, Riyadh, Saudi Arabia between January 2015 and March 2025. Patients were identified through institutional electronic medical records. Eligible patients were aged ≥18 years and had received at least one cycle of ICI therapy as part of routine clinical care. ICIs included anti–PD-1 agents (nivolumab and pembrolizumab), anti–PD-L1 agents (atezolizumab, durvalumab, and avelumab), and anti–CTLA-4 therapy (ipilimumab). Patients with missing key survival data or insufficient follow-up for outcome assessment were excluded. A total of 385 eligible patients were included in the final analysis (**Figure 1**).

### 2.2 Definition and classification of irAEs

Information regarding irAEs were identified through retrospective review of the electronic medical records based on the clinical documentation and assessment of the treating oncology physicians. irAEs were defined as toxicities considered by the treating physicians to be related to ICI therapy after exclusion of alternative etiologies, including infection, disease progression, and concomitant medications. The severity of irAEs was graded according to the Common Terminology Criteria for Adverse Events (CTCAE) version 5.0. Time to irAE onset was defined as the interval between initiation of ICI therapy and the first documented occurrence of the irAE.

### 2.3 Data collection

Clinical and demographic data were retrospectively extracted from the electronic medical records using a standardized data collection form. Collected variables included age, sex, body mass index (BMI), smoking history, Eastern Cooperative Oncology Group (ECOG) performance status, tumor type, disease stage, comorbidities, prior surgery, prior radiotherapy, type of ICI, treatment duration, and laboratory parameters including neutrophil-to-lymphocyte ratio (NLR). Information regarding irAEs was collected from physician documentation. Variables related to irAEs included organ system involvement, severity, time to onset, management strategies, emergency room or intensive care unit admission, corticosteroid use, additional immunosuppressive therapy, treatment interruption or discontinuation, and ICI reintroduction were also collected.

### 2.4 Landmark analysis

To minimize immortal time bias associated with irAE occurrence, separate three-month landmark analyses were performed for OS and PFS. Only patients who remained alive and under follow-up beyond the predefined three-month landmark were included in the OS landmark cohort. For the PFS landmark analysis, patients were additionally required to be progression-free at the three-month landmark. Survival time was calculated from the three-month landmark, which served as the time origin for both the OS and PFS landmark analyses.

Within the landmark cohorts, the primary survival analyses were performed using Kaplan–Meier methods, in which patients were classified according to whether they developed at least one irAE at any time during follow-up. This approach was chosen to evaluate the overall association between irAE development and survival throughout the course of ICI therapy, recognizing that ICIs are administered over multiple treatment cycles and clinically relevant irAEs may develop after several treatment cycles rather than exclusively during the early treatment period. To address the potential for immortal time bias associated with this exposure definition, an additional sensitivity analysis was performed using multivariable Cox proportional hazards regression. For this analysis, irAE exposure was defined exclusively according to events occurring within the first three months after ICI initiation. Patients who experienced at least one irAE during this period were classified as irAE-positive, whereas those without an irAE within the first three months, including patients whose first irAE occurred after the landmark, were classified as irAE-negative.

A total of 8 patients were excluded from the OS landmark analysis because they died before the three-month landmark. For the PFS analysis, 8 patients were excluded because they experienced progression or death before the three-month landmark. In addition, 28 patients were excluded from the PFS analysis because progression data were unavailable. Consequently, the final landmark cohort consisted of 377 patients for the OS analysis and 349 patients for the PFS analysis (**Figure 1**).

### 2.5 Statistical analysis

Continuous variables were summarized using medians and ranges, while categorical variables were presented as frequencies and percentages. Comparisons between categorical variables were performed using the chi-square test or Fisher’s exact test, as appropriate. Continuous variables were compared using the Student’s t-test or Mann–Whitney U test according to data distribution. A landmark analysis was conducted to minimize immortal time bias associated with the occurrence of irAEs. A landmark time of three months following initiation of ICI therapy was predefined, and only patients who remained alive beyond the landmark period were included in survival analyses. OS was defined as the time from the landmark date to death from any cause or last follow-up. PFS was defined as the interval from the landmark date to the first documented disease progression, death from any cause, or last follow-up, whichever occurred first. Disease progression was determined based on the documented radiological and/or clinical assessment of the treating oncology physicians during routine clinical practice. Because of the retrospective design, centralized radiologic review and formal RECIST 1.1 assessment were not performed.

Kaplan–Meier methods were used to estimate OS and PFS, and survival distributions between patients who developed at least one irAE during follow-up and those who did not were compared using the log-rank test. To assess the robustness of these findings while minimizing immortal time bias, separate three-month landmark sensitivity analyses were performed for OS and PFS using multivariable Cox proportional hazards regression.

Candidate variables for the multivariable models were selected based on clinical relevance, and established prognostic importance, and included demographic, clinical, and treatment-related characteristics. The proportional hazards assumption was assessed using Schoenfeld residuals (ASSESS statement in SAS PROC PHREG). Variables violating the proportional hazards assumption were excluded from the corresponding final models. Analyses were performed using a complete-case approach; therefore, observations with missing values for variables included in a given model were excluded from that analysis. Adjusted hazard ratios (HRs) with corresponding 95% confidence intervals (CIs) were reported. All statistical tests were two-sided, and a *P*-value <0.05 was considered statistically significant. Statistical analyses were performed using Statistical Analysis Software (SAS), version 9.4 (SAS Institute Inc., Cary, NC, USA).

### 2.6 Ethical considerations

The study was approved by the Institutional Review Board of King Abdullah International Medical Research Center (KAIMRC). Due to the retrospective nature of the study and use of de-identified clinical data, the requirement for informed consent was waived. All patient data were anonymized prior to analysis to ensure confidentiality and compliance with institutional ethical standards.

## 3 Results

### 3.1 Patient characteristics

A total of 385 patients with solid tumors treated with ICIs were included in the study (**Figure 1**). Among them, 181 patients (47%) developed at least one irAE, while 204 patients (53%) did not develop irAEs during treatment. Baseline clinicopathological characteristics of the study population are summarized in (**Table 1**). The median age was 63 years in the irAE group and 61 years in the non-irAE group (P = 0.076). Male patients represented the majority of the cohort in both groups (68.5% vs. 65.2%, P = 0.491). No significant differences were observed between the groups regarding BMI, smoking status, or baseline comorbidities including hypertension, diabetes mellitus, cardiac disease, and chronic kidney disease. The most common tumor types were hepatobiliary malignancies, lung cancer, and genitourinary cancers. For AJCC staging, most patients had advanced-stage disease, with stage IV tumors accounting for 87.85% of irAE group and 86.93% of non-irAE group included in this cohort. Anti–PD-1 therapy represented the predominant immunotherapy regimen in both groups. There were no statistically significant differences between patients with and without irAEs regarding tumor type, disease stage, ECOG performance status, prior surgery, prior radiotherapy, or type of immunotherapy regimen (**Table 1**).

**Table 1:** Baseline characteristics of ICI-treated patients.

Variables	irAEs n=181 (47%)	No-irAEs n=204 (53%)	p-value
Age Median	63 (55-71)	61 (52-72)	0.076
Male gender	124 (68.51%)	133 (65.20%)	0.4911
BMI (kg/m2)			
Less than 20	44 (24.31%)	51 (25%)	0.8713
20 up to 25	52 (28.73%)	53 (25.98%)	
Over 25 to 30	47 (25.97%)	51 (25%)	
Over 30	38 (20.99%)	49 (24.02%)	
Comorbidities			
Hypertension	103 (56.91%)	110 (53.92%)	0.5566
Diabetes	93 (51.38%)	100 (49.02%)	0.6437
Cardiac disease	23 (12.71%)	13.24%)	0.8777
Chronic kidney disease	21 (11.60%)	23 (11.27%)	0.8050
Smoking (Yes)	33 (18.23%)	28 (13.73%)	0.2268
Tumor type			
Breast Cancer	7 (3.89%)	15 (7.35%)	0.6655
Gastrointestinal (GI) cancer	28 (15.58%)	40 (19.61%)	
Genitourinary (GU) cancer	33 (18.33%)	40 (19.61%)	
Gynecologic Cancer	1 (0.56%)	0	
Head & Neck Cancer	14 (7.78%)	11 (5.39%)	
Hepatobiliary Cancer	43 (23.89%)	41 (20.10%)	
Lung Cancer	32 (17.78%)	40 (19.61%)	
Melanoma	10 (5.56%)	5 (2.45%)	
Multiple Primary Cancers	4 (2.22%)	3 (1.47%)	
Sarcoma	4 (2.22%)	5 (2.45%)	
Skin cancer	0	1 (0.49%)	
Soft Tissue Sarcoma	1 (0.56%)	1 (0.49%)	
Thoracic cancer	2 (1.11%)	2 (0.98%)	
AJCC staging			
Stage 1	2 (1.10%)	–	0.2265
Stage 2	2 (1.10%)	7 (3.52%)	
Stage 3	18 (9.94%)	19 (9.55%)	
Stage 4	159 (87.85%)	173 (86.93%)	
Unknown		5 (missing cells)	
Performance status (ECOG)			
Less that 2	52 (29.38%)	70 (35.35%)	0.2176
2 or more	125 (70.62%)	128 (64.65%)	
Immunotherapy regimen			
anti-PD-1 antibody	166 (91.71%)	177 (86.76%)	0.2790
anti-PD-L1 antibody	12 (6.63%)	23 (111.27%)	
anti-CTLA-4 antibody	3 (1.66%)	4 (1.96%)	
Prior surgery	60 (33.15%)	79 (38.73%)	0.2555
Prior radiotherapy	83 (45.86%)	80 (39.22%)	0.1881

P-value for Numerical comparison were computed using mann-whitney test. for Categorical comparison, chi-square and fisher's exact test were used.

### 3.2 Incidence and clinical characteristics of IrAEs

Among the 385 included patients, 181 (47%) developed at least one irAE during ICI treatment. The severity profile of the observed irAEs is summarized in **Table 2**. Most irAEs were mild to moderate, with Grade I events accounting for 109 (62.6%) cases and Grade II events for 34 (19.5%), whereas Grade III and Grade IV events occurred in 24 (13.8%) and 7 (4.0%) cases, respectively. With respect to timing, 76 (43.18%) irAEs occurred within the first three months of treatment, while 100 (56.82%) occurred thereafter (**Table 2**). The most frequently affected organ systems were hepatic, endocrine, pulmonary, gastrointestinal, and dermatologic toxicities. The most common irAEs included transaminitis (n = 86), thyroiditis (n = 40), pneumonitis (n = 31), rash (n = 26), and colitis (n = 14), whereas cholangitis, hypothyroidism, and hyperthyroidism were less frequently observed (**Table 2**).

**Table 2:** Overall onset, type and severity of irAEs.

irAEs	n – (%)	Median OS 95% CI MONTH	Log-rank P
CTCAE grades			
Grade I	109 (62.64%)	52.09 (39.60 – 69.27)	0.0101*
Grade II	34 (19.54%)	70.45 (54.45 – 74.56)	
Grade III	24 (13.79%)	63.29 (38.29 – ns)	
Grade IV	7 (4.02%)	21.04 (3.86 – ns)	
Time to onset			
≤3 months	76 (43.18%)	53.83 (39.60 – 71.70)	0.9974*
>3 months	100 (56.82%)	63 29 (52.09 – 71.60)	
irAEs type**	n – (%)		
Pneumonitis	31 (17.13%)		
Cholangitis	4 (2.21%)		
Colitis	14 (7.74%)		
Transaminitis	86 (47.51%)		
rash	26 (14.37%)		
Hypothyroidism	5 (2.76%)		
Hyperthyroidism	2 (1.10%)		
thyroiditis	40 (22.10%)		

*Log-rank test were used to compute the P-value.

Patients could experience more than one irAE; therefore, the total number of irAEs exceeds the number of patients.

**Patients may have experienced more than one irAEs; therefore, the total number of irAEs exceeds the number of patients.

### 3.3 Management of IrAEs

Management strategies for irAEs are summarized in (**Table 3**). Emergency room or intensive care unit admission related to irAEs occurred infrequently, involving 11 patients (5.6%) during the first irAE episode and 6 patients (3.0%) during subsequent episodes (**Table 3**). Permanent discontinuation of immunotherapy due to toxicity occurred in 22 patients (11.1%) after the first irAE and in 9 patients (4.5%) following recurrent irAEs (**Table 3**). Corticosteroid therapy was required in a subset of patients, with 24 patients (12.1%) receiving systemic steroids for the first irAE episode and 11 patients (5.5%) requiring steroids for subsequent irAEs. Reintroduction of ICI after toxicity resolution was performed in a limited number of patients. No patients required additional immunosuppressive therapies beyond corticosteroids.

**Table 3:** Management of irAEs.

Management	*n* – (%)
irAE requiring ER/ICU admission	First irAE episode: 11 (5.55%). Second irAE episode: 6 (3.03%) Third irAE episode: None
Discontinuation of ICI Treatment	First irAE episode: 22 (11.11%) Second irAE episode: 9 (4.54%)
Steroids treatment	First irAE episode: 24 (12.12% Second irAE episode: 11 (5.55%)
Immunotherapy Re-introduced	First irAE episode: 5 (2.52%) Second: irAE episode 3 (1.51%)
Required other immunosuppression	First irAE episode: 0 Second irAE episode: 0

### 3.4 OS outcome

**Figure 2:**
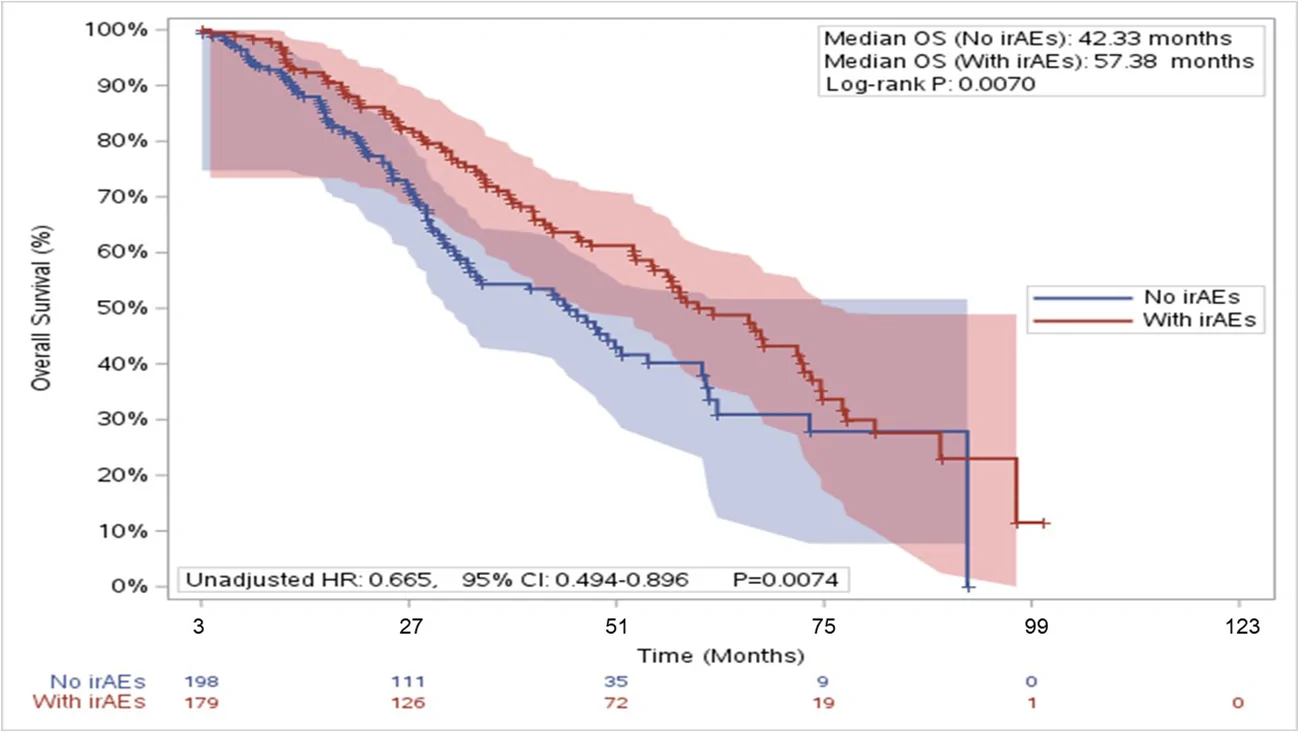
Kaplan–Meier OS curves according to the development of irAEs. Kaplan–Meier curves comparing OS between patients who developed irAEs and those who did not. A three-month landmark analysis was performed to minimize immortal time bias; therefore, only patients who remained alive and under follow-up beyond the three-month landmark were included (n = 377). OS was measured from the landmark date to death from any cause or last follow-up. The shaded areas represent the 95% confidence intervals. Unadjusted hazard ratios (HRs) were estimated using Cox proportional hazards regression, and survival distributions were compared using the log-rank test.

Kaplan–Meier survival analysis demonstrated significantly improved OS among patients who developed irAEs compared with those who did not develop irAEs. To minimize immortal time bias, a three-month landmark analysis was performed. Median OS was 57.38 months in the irAE group compared with 42.33 months in the non-irAE group (log-rank P = 0.0070) (**Figure 2**). In a multivariable Cox proportional hazards analysis, in which irAE exposure was defined by events occurring within the first three months after ICI initiation and adjusted for clinically relevant covariates, development of irAEs remained associated with improved OS (HR 0.673, 95% CI 0.453–1.00; P = 0.0498) (**Table 4**). Baseline NLR <3 was also associated with favourable OS (HR 0.603, 95% CI 0.439–0.829; P = 0.0018) (**Table 4**).

**Table 4:** Adjusted overall survival HR for time-dependent AE and survival 3 months- landmark analysis.

Variables	HR 95% CI	P-Value
irAE (≤3 months vs. >3 or no irAE)	0.673 (0.453 – 1.00)	0.0498
ICI (Yes vs. No)	0.892 (0.648 – 1.228)	0.4845
Age	1 (0.991 – 1.009)	0.9608
Diabetes (Yes vs. No)	1.174 (0.802 – 1.720)	0.4087
Hypertension (Yes vs. No)	0.822 (0.559 – 1.207)	0.3174
Baseline NLR < 3 (Yes vs. No)	0.603 (0.439 – 0.829)	0.0018
Surgery (Yes vs. No)	0.871 (0.618 – 1.226)	0.0863
Smoke (Yes vs. No)	1.100 (0.723 – 1.673)	0.6564
Cardiac Disease (Yes vs. No)	1.001 (0.606 – 1.655)	0.9959
Radiotherapy (Yes vs. No)	0.951 (0.677 – 1.334)	0.7698
Immunotherapy Drug (anti-PD-L1 antibody vs anti-PD-1 antibody)	0.632 (0.327 – 1.221)	0.1718
Immunotherapy Drug (anti-CTLA-4 antibody vs anti-PD-1 antibody)	2.599 (1.017 – 6.642)	0.0460
Staging AJCC (1 vs 4)	2.080 (0.449 – 9.632)	0.3492
Staging AJCC (2 vs 4)	2.578 (1.012 – 6.567)	0.0471
Staging AJCC (3 vs 4)	1.735 (1.037 – 2.903)	0.0359
ECOG (0 vs 5)	1.122 (0.542 – 2.326)	0.7564
ECOG (1 vs 5)	1.169 (0.659 – 2.074)	0.5940
ECOG (2 vs 5)	1.836 (1.042 – 3.236)	0.0357
ECOG (3 vs 5)	1.673 (0.929 – 3.011)	0.0863
ECOG (4 vs 5)	1.052 (0.603 – 1.836)	0.8580

The proportional hazards assumption was assessed using Schoenfeld residuals with a resampling-based test and was not violated. Tumor type and gender were excluded because they violated the assumption; no violation was detected among the retained variables presented in the table. Model fit was evaluated using the likelihood ratio test resulting in statistically significant based on the test.

### 3.5 PFS outcome

**Figure 3:**
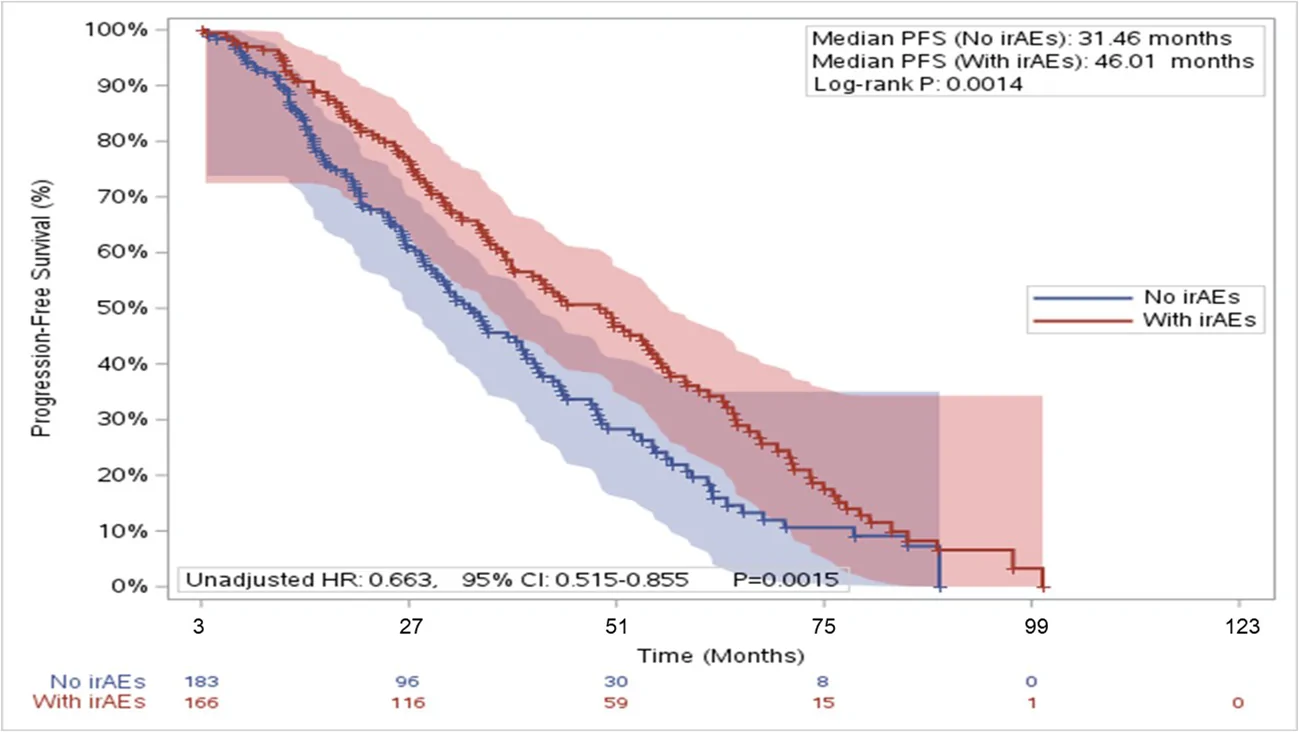
Kaplan–Meier PFS curves according to the development of irAEs. Kaplan–Meier curves comparing PFS between patients who developed irAEs and those who did not. A three-month landmark analysis was performed to minimize immortal time bias; therefore, only patients who remained alive, progression-free, and under follow-up beyond the three-month landmark were included (n = 349). PFS was measured from the landmark date to disease progression, death, or last follow-up, whichever occurred first. The shaded areas represent the 95% confidence intervals. Unadjusted hazard ratios (HRs) were estimated using Cox proportional hazards regression, and survival distributions were compared using the log-rank test.

Patients who developed irAEs also demonstrated significantly improved PFS compared with patients without irAEs. To minimize immortal time bias, a three-month landmark analysis was performed. Median PFS was 46.01 months in the irAE group compared with 31.46 months in the non-irAE group (log-rank P = 0.0014) (**Figure 3**). In a multivariable Cox proportional hazards analysis, in which irAE exposure was defined by events occurring within the first three months after ICI initiation and adjusted for clinically relevant covariates, early irAE occurrence was not independently associated with PFS (HR 0.851, 95% CI 0.630–1.149; *P* = 0.2916) (**Table 5**). In contrast, a baseline NLR <3 remained an independent predictor of favorable PFS (HR 0.656, 95% CI 0.503–0.856; *P* = 0.0019) (**Table 5**).

**Table 5:** Adjusted progression-free survival HR for time-dependent AE and survival 3 months- landmark analysis.

Variables	HR 95% CI	P-Value
irAE (<3 months vs. > 3 and no irAE)	0.851 (0.630 – 1.149)	0.2916
ICI (Yes vs. No)	0.833 (0.638 – 1.089)	0.1817
Hypertension (Yes vs. No)	0.973 (0.740 – 1.280)	0.8443
Baseline NLR < 3 (Yes vs. No)	0.656 (0.503 – 0.856)	0.0019
Smoke (Yes vs. No)	1.037 (0.732 – 1.469)	0.8390
Cardiac Disease (Yes vs. No)	1.124 (0.745 – 1.696)	0.5775
Radiotherapy (Yes vs. No)	0.939 (0.717 – 1.230)	0.6487
Staging AJCC (1 vs 4)	17.828 (2.350 – 135.267)	0.0053
Staging AJCC (2 vs 4)	1.775 (0.722 – 4.364)	0.2114
Staging AJCC (3 vs 4)	1.329 (0.831 – 2.125)	0.2357

The proportional hazards assumption was assessed using Schoenfeld residuals with a resampling-based test and was not violated. Tumor type, Age, ECOG, Surgery, Diabetes and gender were excluded because they violated the assumption; no violation was detected among the retained variables presented in the table. Model fit was evaluated using the likelihood ratio test resulting in statistically significant based on the test.

## 4 Discussion

This study evaluated the association between irAEs, and clinical outcomes in 385 patients with solid tumors treated with ICIs. To minimize immortal time bias, OS and PFS were analyzed using a predefined three-month landmark approach, whereby survival time was calculated from the three-month landmark. Within this landmark cohort, patients who developed at least one irAE during follow-up experienced significantly improved OS and PFS compared with those who did not develop irAEs. We additionally performed multivariable Cox regression using irAE occurrence within the first three months as the exposure variable to further evaluate the association between early irAE development and survival outcomes. In this analysis, early irAE occurrence remained independently associated with improved OS, whereas the association with PFS was no longer statistically significant. Baseline NLR <3 also emerged as an independent predictor of favorable outcomes. Collectively, these findings suggest that irAE development and baseline systemic inflammatory status may be associated with improved clinical outcomes in patients receiving ICIs. However, because of the retrospective observational design, these associations should be interpreted with caution and cannot establish causality.

Our finding are consistent with growing body of evidence suggesting that patients who develop irAEs have better clinical benefit from ICIs (27). However, interpretation of these findings remains challenging because many studies are susceptible to immortal-time bias, whereby patients must survive long enough to develop toxicity. Failure to account for this time-dependent phenomenon may exaggerate the clinical benefit associated with irAEs (28). By incorporating a predefined three-month landmark design and an additional multivariable analysis restricted to early irAEs, our study sought to minimize this bias. The persistence of the association with OS, together with the attenuation of the PFS association after multivariable adjustment, suggests that the relationship between irAEs and survival is complex and should be interpreted cautiously. Future prospective studies incorporating time-dependent analytical approaches are warranted to better define the prognostic significance of irAEs.

In addition to irAEs, baseline NLR emerged as an independent prognostic factor in our cohort. NLR has been reported as biomarker of systemic inflammation and host immune status (29, 30) and associated with an immunosuppressive tumor microenvironment, increased neutrophil-mediated tumor progression, impaired lymphocyte-mediated anti-tumor immunity, and poorer clinical outcomes across multiple malignancies (31, 32). Numerous studies evaluating patients treated with ICIs have also demonstrated that elevated NLR is associated with inferior survival (33). Our findings further support the prognostic value of baseline inflammatory status and suggest that NLR may complement irAE occurrence in identifying patients more likely to derive benefit from immunotherapy.

In this study, the incidence of irAEs was 47%, where the incidence of any-grade irAEs generally ranges in the literature from approximately 30% to 50% depending on tumor type, ICI regimen, and study population (34). The type of irAEs also aligns with the literature, with hepatic, endocrine, pulmonary, gastrointestinal, and dermatologic irAEs representing the most frequently reported irAEs (35), while pneumonitis and colitis associated with later onset after ICI treatment. Most irAEs in our cohort were low grade, whereas severe toxicities were relatively uncommon. These findings further highlight the heterogeneous and multisystem nature of irAEs during ICI treatment.

Our study also reported on the management of irAEs. For example, Corticosteroids represented the primary treatment strategy for irAEs, whereas intensive care admission and permanent treatment discontinuation were relatively uncommon. Although the impact of corticosteroids on ICI efficacy remains controversial (36–38), the present study was not designed to evaluate the impact of corticosteroid exposure on ICI efficacy. Detailed information regarding steroid dose, duration, timing, and indication was not consistently available; therefore, no conclusions can be drawn regarding the relationship between corticosteroid therapy and survival outcomes. Future prospective studies with comprehensive corticosteroid exposure data are needed to better define this association.

Several aspects distinguish our study from existing literature. First, unlike many retrospective analyses, we employed a landmark approach specifically designed to eliminate immortal time bias. Second, we evaluated the prognostic impact of irAEs and baseline inflammatory status within the same multivariable framework, demonstrating that both factors independently contribute to clinical outcomes. Third, our cohort included a heterogeneous population of solid tumors with substantial representation of hepatobiliary, gastrointestinal, genitourinary, and lung malignancies, extending the evidence beyond the melanoma- and NSCLC-dominated populations commonly reported in the literature. Finally, our study provides data from a Middle Eastern population, a region that remains underrepresented in immunotherapy outcome research (25), while also incorporating detailed characterization of irAE management and treatment modifications.

Several limitations should be acknowledged. First, the retrospective single-canter design may introduce selection bias and limit generalizability. Second, retrospective identification of irAEs is dependent on the accuracy and completeness of physician documentation and may have resulted in underreporting of low-grade toxicities. Third, although we performed a predefined landmark sensitivity analysis to address immortal-time bias, residual bias cannot be completely excluded, and the retrospective observational design precludes causal inference. Finally, the study was not adequately powered to evaluate whether the association between irAEs and survival differed according to individual organ systems or the cumulative number of irAEs. The relatively small number of patients within several organ-specific irAE categories limited meaningful subgroup analyses and would likely have produced unstable estimates.

In conclusion, the development of irAEs was associated with improved survival in patients with solid tumors treated with ICIs, and this association remained generally consistent in a landmark sensitivity analysis designed to minimize immortal-time bias. Baseline NLR also emerged as an independent predictor of survival outcomes. Together, these findings suggest association between the development of irAEs and improved clinical outcomes, while highlighting the prognostic value of baseline systemic inflammatory status. However, because of the retrospective observational design, these associations should be interpreted cautiously and should not be considered evidence of causality. Future prospective multicenter studies are warranted to further elucidate the biological and clinical significance of irAEs during ICI treatment.

## Data Availability

The original contributions presented in the study are included in the article/supplementary material. Further inquiries can be directed to the corresponding author.
